# A detailed analysis of pediatric surgical malpractice claims in Germany: what is the probability of a pediatric surgeon to be accused or convicted?

**DOI:** 10.1007/s00423-020-02069-6

**Published:** 2021-01-08

**Authors:** Sara Mahler, Emilio Gianicolo, Oliver J. Muensterer

**Affiliations:** 1 Department of Pediatric Surgery, University Medical Center of the Johannes Gutenberg University Mainz, Mainz, Germany; 2 Institute of Medical Biostatistics, Epidemiology and Informatics, University Medical Center of the Johannes Gutenberg University Mainz, Mainz, Germany; 3 Institute of Clinical Physiology of the Italian National Research Council, Lecce, Italy; 4 Department of Pediatric Surgery, Dr. von Hauner Children’s Hospital, University Medical Center of the Ludwig-Maximilians-University of Munich, Lindwurmstrasse 4, 80337 Munich, Germany

**Keywords:** Pediatric surgery, Malpractice, Litigation, Conviction, Germany

## Abstract

**Aim of the study:**

Pediatric surgeons treat a vulnerable population in which unfavorable outcome can lead to substantial long-term costs, placing them at risk for malpractice claims. This study aims to characterize the frequency and circumstances in which malpractice claims were successfully brought against pediatric surgeons in Germany over the last 5 years.

**Materials and methods:**

Anonymous data on medical treatment errors and payments were acquired from the Federal Chamber of Physicians from 2014 through 2018 and analyzed for most frequent diagnoses and circumstances that resulted in accusation or conviction. Those claims that were successfully rebutted were compared to as controls. Lifetime risk for being involved in litigation and its outcome was calculated.

**Results:**

There were 129 medical malpractice claims over the 5-year observation period. Medical error was confirmed in 56 cases (43%); the rest were successfully appealed. The risk of the prototypical German pediatric surgeon to be accused was 5.24% and to be convicted 2.27% per year in practice. The most common reasons for conviction (alone or in combination) were surgical-technical errors (23%), treatment delay (21%), insufficient workup (17%), incorrect diagnosis (17%), and incomplete consent (16%).The most frequent circumstances leading to a conviction were trauma (27%), inguinal hernia (7%), circumcision (7%), testicular torsion (7%), acute abdomen (7%), and appendicitis (5%).

**Conclusion:**

Over a 40-year career, pediatric surgeons in Germany face an average calculated risk of 2.1 to be accused and 0.9 to be convicted of malpractice claims. Certain circumstances pose higher risks for litigation than others. Knowledge of these patterns may help practitioners avoid medicolegal confrontation.

## Introduction

Although the number of malpractice claims has reportedly been relatively stable in almost every specialty and around the world, the size of monetary payments has increased dramatically over the last years and it seems that this trend will continue [1]. The reasons for this development are unknown, but information, globalization, increased awareness on behalf of patients and family, as well as access to the legal system may play a role.

Physicians are among the most commonly sued professionals in the world [2]. The implications of this development includes the practice of “defensive medicine,” ordering or performing unnecessary tests and procedures, which increases healthcare costs and diminishes resources available for other areas of patient care [3]. In the USA alone, it is estimated that defensive medicine contributes to an additional $60 billion in healthcare expenditures each year [3]. Being accused or sued for medical malpractice also impacts negatively on work quality and satisfaction.

Surgeons in general are particularly vulnerable to being involved in malpractice litigation [4]. Pediatric surgeons treat an extremely vulnerable population, in which unfavorable outcome can lead to substantial long-term costs, placing them at an even higher risk for malpractice claims [1, 5].

To date, there are sparse data on malpractice claims in the field of pediatric surgery, especially in Germany [6]. Many physicians do not have adequate understanding of the risks and implications involved [7]. This study analyzes the centrally filed malpractice claims in pediatric surgery over a 5-year period, evaluates risk factors, circumstances and outcomes, and thereby aims to draw conclusions for clinical risk management in the future.

## Materials and methods

A query on malpractice claims over a time period of 5 years including 2014 through 2018 was retrospectively performed within the database of the German Bundesärztekammer (the Federal Chamber of Physicians). This was performed across all specialties in general, and pediatric surgery in particular. The query included all states except Bavaria, which does not participate in this database. Demographics of the claimants, in- and outpatient treatment status, diagnoses leading to an accusation, circumstances and types of errors, as well as liability outcome parameters were included in the analysis. Also, interdisciplinary claims were identified, in which other specialties besides pediatric surgery were accused. Diagnoses were extracted using Diagnosis Related Groups (DRG) admission codes. Mechanisms and circumstances were extracted by reading anonymous full-text of the verdicts. Data was presented descriptively. Conviction rates according to in- or outpatient status and academic versus non-academic center were compared using the chi-square test. The probabilities to be accused or convicted of medical malpractice were modeled from demographic national practice data. Since only anonymous data was provided and processed, ethics board approval was not necessary.

## Results

**Fig. 1:**
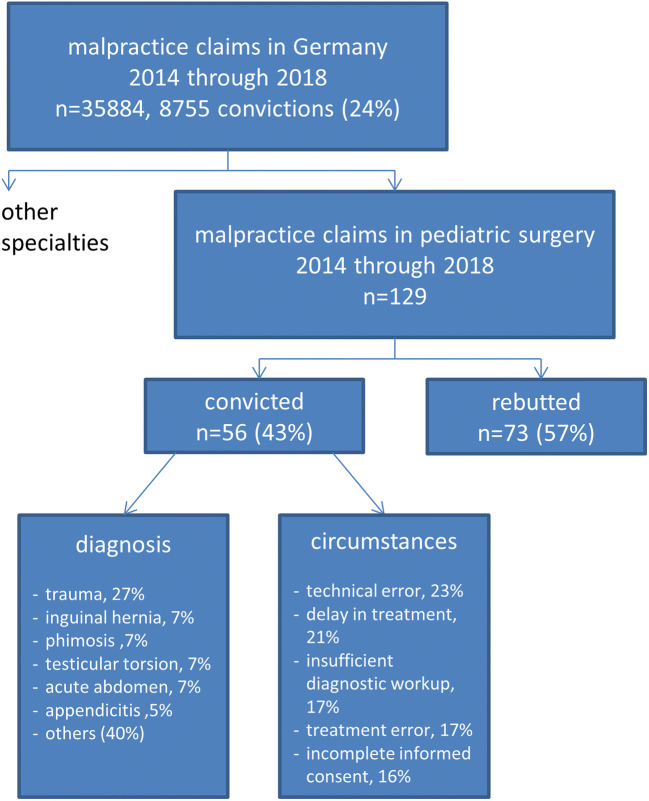
Flowchart of malpractice cases in Germany over a 5-year period

In the 5-year observation period, a total of 35,884 malpractice claims were brought forward in Germany, excluding the state of Bavaria. Of these, 129 (0.36%) were filed against pediatric surgeons. In general, across all specialties, 8755 cases (24% of all accusations) led to conviction, as did 56 cases (43% of all accusations) in the field of pediatric surgery. All others were rebutted successfully (Fig. 1).

Patients involved in the pediatric surgical cases were newborns up to 18 years with a median of 6 years of age. There were 81 male and 48 female patients. The claims resulted from treatments that were performed from 2006 up to 2018. There was an average time interval of 3 years between treatment and verdict.

The most common diagnoses leading to a conviction of malpractice in pediatric surgery were trauma (*n* = 15, 27%), followed by inguinal hernia, phimosis, testicular torsion and acute abdomen (*n* = 4, 7% each), and appendicitis (*n* = 3, 5%). Rarer diagnoses were urachal remnant, teratoma, bronchiolitis, myelomeningocele, gastroschisis, varicocele, hypothyroidism, and Hirschsprung disease.

The most common circumstances ending up in medicolegal liability in Pediatric Surgery—alone or in combination—where technical errors during surgery (*n* = 13, 23%), delay in treatment (*n* = 12, 21%), insufficient diagnostic workup (*n* = 10, 17%), incorrect diagnosis (*n* = 10, 17%), error in treatment (*n* = 10, 17%), and incomplete informed consent (*n* = 9, 16%).

In 14 of the 129 accusations, other specialties were also named, including neonatology, anesthesiology, vascular surgery, and urology. Four of these interdisciplinary cases resulted in conviction.

Twenty-three of the total cases were accusations that resulted from ambulatory outpatient treatment (of these, 13 were appealed, 10 convictions), while 106 resulted from inpatient treatment (of these, 60 were appealed, 46 convictions). There was no difference between out- and inpatient treatments by chi-square analysis (*p* = 0.82). There was also no difference in the distribution between cases in academic (*n* = 31, 18 appealed, 13 convictions) and non-academic centers (*n* = 98, 55 appealed, 43 convictions; *p* = 0.85).

According to the German Federal Chamber of Physicians, there were an average of 607 pediatric surgeons in practice during the study interval in Germany, while according to the State Chamber of Physicians of Bavaria (Bayerische Landesärztekammer), 115 of these were in active practice there, leaving a total of 492 pediatric surgeons in the corresponding other 15 states from which malpractice data are available. Thus, assuming a total of 2460 person-years, the calculated likelihood of being accused of malpractice according to this model is 5.24 per 100 person-years, while the chance of being convicted is 2.27 per 100 person-years in Germany excluding Bavaria. This means that over a 40-year career, pediatric surgeons in Germany face an average calculated risk of 2.1 (on average, once every 19 years) to be accused and 0.9 (once every 44 years) to be convicted of malpractice.

## Discussion

Although the absolute number of malpractice claims in pediatric surgical specialties seems to have remained stable over the last several years [8, 9], these incidences have severe consequences on how medicine and surgery are practiced. Also, the level of indemnity payment has increased in the last years, representing a substantial cost for healthcare systems [4]. Surgeons, and particularly pediatric surgeons, are at a high risk for medicolegal liability. Our study demonstrated that although pediatric surgeons make up only 0.16% of the physician workforce in Germany, 0.36% of all malpractice claims are filed against them (a relative risk of 2.25).

In Germany, accusations of malpractice are initially filed with the medical board of the respective state in which the patient was treated. There, reconciliation between the parties is attempted through an arbitration committee, with the help of external consultants as needed. If this process of mediation fails, the case goes on to court. The cases included in this study’s rebuttal group included all those in which no wrongdoing was ultimately found, either through the state medical board arbitration process, or by court verdict.

Since the database used for this study does not include the monetary payments and compensations resulting from the lawsuits, we cannot comment on the impact on insurance premiums. Practicing defensive medicine may also decrease efficacy of a practitioner and increase healthcare costs. In fact, a recent study found that 1000 surveyed neurosurgeons said they had to order unnecessary tests and radiological examinations in an effort to reduce the perceived risk of medical malpractice claims [3]. Generally, due to the long-term consequences of medical errors, the monetary penalties and compensations resulting from litigation that involves children are higher than those that involve adults [1, 10–12]. This is a finding that was noted in other pediatric subspecialties such as pediatric urology, oromaxillofacial surgery, orthopedics, and in other healthcare settings as well [13–15]. In a recent systematic review of pediatric orthopedic cases in the USA, over half of cases ultimately resulted in indemnity payments [16]. Some technical approaches, such as pediatric minimally invasive surgery, may be more prone to malpractice accusations as well [17].

Besides economics, being accused of medical wrongdoing can cause severe psychological and emotional stress [5, 10]. Questioning self-worth as a practitioner after a conviction for medical practice can lead to burnout [2, 18]. Considering that every pediatric surgeon can count on facing a malpractice claim at least once during their career, it would seem reasonable to include coping strategies for these kinds of difficult situations in specialty training curricula.

Another finding of our study is that in absolute numbers, routine “bread and butter” cases are most likely to lead to litigation. Naturally, this is primarily a function of frequency, since cases such as inguinal hernia repair or appendectomy are simply the most prevalent procedures. This has been described by others, and in other subspecialties as well [3, 15, 19]. A recent study that queried a national database on malpractice cases in the USA also found that technical complications were the most common allegation against surgeons, followed by cases that involved a delayed or missed diagnosis [20]. Similar to our findings, 39% of cases in that study were successfully rebutted. The implication for the practitioner is that they should never be overcome by complacency: Even simple, routine procedures can result in malpractice claims, and technical mistakes are often the basis for litigation. Therefore, good communication, meticulous technique and attention to detail, as well as an open, transparent and close relationship to the patients and their caregivers should always be maintained, no matter how trivial a case may appear.

Complex cases that require an interdisciplinary approach may result in proceedings against several specialties of the same institution. In our series, this was the case in roughly 10% of overall medicolegal cases, consistent with other studies [14, 21–23]. Often, deficits in interdisciplinary communication between members of different departments were a primary factor for the litigation. Another factor was mixed messages given to the families and caregivers by co-treating specialists. Therefore, we believe that interdisciplinary and interpersonal communication skills, along with strategies on how to coordinate the transmission of information to families and caregivers in complex situations should be a formal part of postgraduate training in pediatric surgery.

One of the most detailed reports of malpractice suits in children treated for fractures was published in 2009 by Vinz and Neu [24]. In their analysis, the authors found a total of 189 cases brought forward to the North German arbitration board during an 8-year time interval from 2000 through 2007. The high number of cases in this study corroborates our findings that trauma is the most important reason for physician litigation when treating children. Interestingly, 64% of the accusations led to conviction, a rate much higher than what we found in our study. This is most likely due to the fact that only pediatric trauma cases were included, which again highlights the higher litigation risk when treating fractures in children. Unfortunately, the authors do not calculate the overall number of physicians that actively treated pediatric fractures in the respective catchment area. Therefore, relative risks for accusation and conviction cannot be calculated from the information in their manuscript.

The limitations of our study include the retrospective design and the anonymous data acquisition that kept us from obtaining exact dates on treatment, filing the complaint, and final verdict. German data privacy protection laws do not allow the transmission of exact dates that later can be used for identification of specific cases or persons.

Also, due to the fact that in many instances, children are treated in cooperation with other specialties such as general surgery and pediatrics, we attempted to obtain the corresponding statistics for these specialties from the Bundesärztekammer as well. Unfortunately, this request was denied by the legal department of the Bundesärztekammer.

Finally, the state of Bavaria does not enter their malpractice claims into the federal database. Unfortunately, we were once again unsuccessful to obtain the corresponding data on malpractice cases from Bavaria. Therefore, only the situation in the other 15 states in Germany could be assessed in this study. Since the Bayerische Landesärztekammer provided us with the number of actively practicing pediatric surgeons in Bavaria, we were able to exactly determine the denominator for modeling the judicial risk for the rest of the country.

## Conclusion

Pediatric surgeons can expect to be involved in medicolegal confrontation at least once during their career. Routine pediatric surgical indications such as trauma care, inguinal hernia repair, and appendectomy make up the bulk of cases that result in litigation. Technical errors during surgery, delay in treatment, insufficient diagnostic workup, wrong diagnosis, and an incomplete informed consent are the main grounds on which pediatric surgeons get convicted. Strategies to decrease the risk of being involved in a court case should include obtaining outside help during surgery for cases that one is not completely familiar with, adherence to published practice guidelines in patient workup and management, and making sure the informed consent process is well and concisely documented in the medical record of the patient.

To decrease the risk of litigation, we suggest incorporating the following action items into routine daily practice:

- Document all interactions with patients in a concise, professional, objective manner.
- Know your limits and practice within these.
- Get help if you face an unfamiliar diagnosis, situation, or operation.
- Involve other colleagues early if problems are suspected.
- Always maintain a transparent and open communication with patients and families, especially if a case did not evolve as expected.
- Establish a proactive risk management system in your department or practice.
